# Amino acid and fatty acid profiles of perennial Baki™ bean

**DOI:** 10.3389/fnut.2023.1292628

**Published:** 2024-01-12

**Authors:** Evan B. Craine, Spencer Barriball, Muhammet Şakiroğlu, Tessa Peters, Brandon Schlautman

**Affiliations:** ^1^The Land Institute, Salina, KS, United States; ^2^Adana Alparslan Türkeş Science and Technology University, Adana, Türkiye

**Keywords:** perennial grain crops, fatty acid profile, amino acid profile, Onobrychis, sainfoin, perennial Baki™ bean

## Abstract

To realize the potential of sainfoins to contribute to sustainable agriculture and expand on demonstrated uses and benefits, *de novo* domestication is occurring to develop perennial Baki™ bean, the trade name used by The Land Institute for pulses (i.e., grain legumes) derived from sainfoins. The objective of this study was to characterize amino acid and fatty acid profiles of depodded seeds from commercial sainfoin (*Onobrychis viciifolia*) seed lots, and compare these results with data published in the Global Food Composition Database for Pulses. The fatty acid profile consisted primarily of polyunsaturated fatty acids (56.8%), compared to monounsaturated (29.0%) and saturated fatty acids (14.2%), and *n*-3 fatty acids (39.5%), compared to *n*-9 (28.4%) and *n*-6 (17.6%) fatty acids. The essential fatty acid linolenic acid (18,3 *n*-3) was the most abundant fatty acid (39.2%), followed by oleic acid (18,1 cis-9) (27.8%), and the essential fatty acid linoleic acid (18,2 *n*-6) (17.3%). The amino acid profile consisted primarily of the nonessential amino acids glutamic acid (18.3%), arginine (11.6%), and aspartic acid (10.8%), followed by the essential amino acids leucine (6.8%), and lysine (5.8%). Essential amino acid content met adult daily requirements for each amino acid. This indicates that sainfoin seeds may be a complete plant protein source. However, further research is necessary to better understand protein quality, defined by protein digestibility in addition to the amino acid profile. By demonstrating favorable fatty acid and amino acid profiles to human health, these results contribute to a growing body of evidence supporting the potential benefits of perennial Baki™ bean, a novel, perennial pulse derived from sainfoins.

## 1 Introduction

The Food and Agriculture Organization (FAO) of the United Nations defines pulses as grain legumes. These crops have a long history as foundational components of global agricultural and food systems (1). By converting atmospheric nitrogen into ammonia via symbiosis with rhizobia soil bacteria (2), legumes are ultimately linked to providing almost the entire amount of nitrogen that livestock and humans must obtain from diets (3, 4). This process allows pulses to accumulate twice the amount of protein of cereal grains (5). Pulses are uniquely positioned as a globally important staple food due to their ability to deliver a complete nutritional package. In addition to providing a sustainable and affordable source of protein, they are a vital source of dietary fiber, slowly digested carbohydrates, vitamins, minerals, and polyphenolics (6, 7). A well-established body of evidence links pulse consumption to a reduced risk of mortality from all causes (8–10). Yet, despite the numerous benefits of pulses to agricultural and food systems, and their potential to address myriad challenges facing agriculture and human health, pulses suffer from low adoption and are underutilized (11, 12).

In addition to these major pulse crops, there are many legume species of minor global economic importance that hold major agricultural importance as food and fodder for humans and animals as part of regional crop and food systems. However, most of these legume species are not well known outside of their primary production regions, where genetic diversity is maintained and improved through farmer-maintained landraces (13, 14). In the context of legumes, this leads to neglected and underutilized, or orphan, status, despite the ability to adapt to specific, often challenging, agroecological conditions and provide nutritional security (15–18).One such example of neglected and underutilized legumes are species in the *Onobrychis* genus (hereafter sainfoins). Sainfoins are temperate perennial legumes originating from central Asia with great potential for sustainable agriculture (19, 20). Sainfoins are undergoing *de novo* domestication at The Land Institute (Salina, KS, US) to develop Perennial Baki™ bean, the trade name used by The Land Institute for pulses derived from *Onobrychis* spp., as a perennial grain legume crop to expand on the demonstrated benefits and uses of sainfoins (21). Unlike all other pulses, which typically include annual species, sainfoins do not require replanting each year. Therefore, sainfoins, like other perennial grain candidates, provide continuous living cover and nitrogen fixation to improve soil health and reduce soil erosion (22–25). Throughout this paper, sainfoin is used to refer to the crop plant, while perennial Baki™ bean is used to refer to the grain legumes (i.e., pulses) derived from sainfoins.

This study is part of an ongoing effort to investigate the quality and safety of Baki™ bean for human consumption. Previously, we showed that Baki™ bean had protein content similar to soybean and lupin, fat content similar to chickpea, high dietary fiber and phytic acid content, and iron and zinc content comparable to most pulses (26). Several studies have also investigated seed composition within the genus *Onobrychis*. Notably, Tarasenko et al. (27), Ditterline (28), and Baldinger et al. (29) quantified amino acids, while Bagci et al. (30), Bakoglu et al. (31), Kaplan et al. (32), and Karataş et al. (33) quantified fatty acids. Complimenting these studies are monogastric animal feeding trials, which demonstrate the potential value of sainfoin seeds in diets of weenling pigs (29) and rats (34). To advance our previous work, the aim of this study was to characterize amino acid and fatty acid profiles in the context of data published in the Global Food Composition Database for Pulses (35).

## 2 Materials and methods

### 2.1 Seed material

Commercial seed companies and/or seed producers from Montana, US provided samples of named sainfoin (*Onobrychis viciifolia*) varieties for analysis (Table 1). Plants were harvested in either 2018 or 2020, in July or August based on individual company and producer schedules. While specific harvest times may vary, seeds are generally harvested when most seeds have reached physiological maturity. This ensure that the greatest quantity of high-quality seed is available for sale into the forage industry to establish new fields. Seed company and producer identities are not disclosed for privacy purposes. The seed samples (*N* = 9) include the sainfoin varieties AAC Mountainview (36), Delaney, Eski (37), Shoshone (38), Renumex (39), and Rocky Mountain Remont. Rocky Mountain Remont is a selection from Remont.^1^ See USDA NRCS Plant Materials Technical Note No. MT-91^2^ for additional information on selected variety releases.

**Table 1:** Baki™ bean sample ID, grower code, variety code, variety, and year (*N* = 9).

ID	Grower Code	Variety Code	Variety	Year
R-S-18	R	S	Shoshone	2018
R-D-18	R	D	Delaney	2018
R-R-18	R	R	Rocky Mountain Remont	2018
W-D	W	D	Delaney	2020
W-R	W	R	Rocky Mountain Remont	2020
W-M	W	M	AAC Mountanview	2020
W-Rx	W	Rx	Renumex	2020
CS-E	CS	E	Eski	2020
CS-S	CS	S	Shoshone	2020

### 2.2 Sample preparation

Before analysis, Baki™ beans were removed from pods (i.e., depodded) using a Halstrop bench top dehuller. Following dehulling, a 3.571 mm sieve was used to separate the pods (i.e., hulls) and seeds. Then, a 2.778 mm sieve was used to separate the seeds into two fractions. The fraction that remained on the sieve was reserved for analysis of the whole seed (i.e., cotyledons and seed coat intact). Approximately 500 g of seed were haphazardly sampled from the total amount of seed available for analysis of each seed sample. All analyses were performed by Great Plains Analytical Laboratories (GPAL) (Kansas City, MO, US) unless otherwise noted. The GPAL quality assurance system is in accordance with International Electrotechnical Commission (ISO/IEC) 17,025:2018 and the Association of Official Agricultural Chemists (AOAC) Requirements for Food and Pharmaceutical Testing Laboratories.

### 2.3 Determination of fatty acid profiles

The Baki™ bean fatty acid profile was determined according to AOAC 996.06 with a detection limit of 0.003% (40). Briefly, the procedure consists of hydrolytic extraction, followed by methylation and analysis of the resulting fatty acid methyl esters (FAMEs) via capillary gas chromatography coupled with flame ionization detection.

### 2.4 Determination of amino acid profiles

The Baki bean™ amino acid profile was determined as described in Schuster (41), with a detection limit of 10 mg/100 g sample. Briefly, two different reagents were used to derivatize primary and secondary amino acids, before separation on a reverse phase column and detection using a diode array detector. Amino acid content was adjusted to mg/g protein by dividing by crude protein content, which has been previously reported for each sample by Craine et al. (26).

### 2.5 External datasets

To compare the Baki bean™ amino acid and fatty acid profiles analyzed using the methods described above to other pulse crop species, data for pulse crop species were downloaded from the Food and Agriculture Organization/International Network of Food Data Systems (FAO/INFOODS) Global Food Composition Database for Pulses (Version 1.0 - uPulses1.0–2017) (35). Data for raw seeds were reported on an edible portion, dry matter content basis. For comparisons, data for the following pulses were selected *Cicer arietinum* (L.) (chickpea), *Lens culinaris* (Medik) (lentil), *Phaseolus vulgaris* (L.) (common bean), *Pisum sativum* (L.) (pea), *Vicia faba* (L.) (broad bean or fava bean) *Vigna radiata* (L.) R Wilczek (mung bean), and *Vigna unguiculata* (L.) Walp (cowpea).

Data for *Glycine max* (L.) (soybean) was downloaded from by the United States Department of Agriculture (USDA) Agricultural Research Service (ARS) FoodData Central (42).

### 2.6 Statistical analyses

When not already present in this form, data were adjusted to a dry matter basis using moisture content. All values, unless otherwise noted, are reported on an edible portion dry matter basis (EPDM). All statistical analyses, unless otherwise noted, were performed using the R statistical software (43). The summarise function (44) or functions in base R were used to generate summary statistics (e.g., count, mean, standard deviation). The standard error of the mean was calculated and reported along with mean values. To test the null hypothesis that the crop species did not differ significantly with respect to the content of each individual, a Kruskal Wallis test was performed for each analyte. *Post hoc* analysis consisted of pairwise comparisons between crop species to determine whether mean values significantly differed, which was performed using Fisher’s least significant difference test with Bonferroni corrected *p* values. The Kruskal Wallis tests and Fisher’s least significant difference tests were performed using the agricolae package in R (45).

## 3 Results

### 3.1 Fatty acid profile

Fatty acid profiles for the sainfoin varieties are provided in Table 2. The content of 45 individual fatty acids was determined, representing the various fatty acids groups. These include saturated fatty acids (SFA), monounsaturated fatty acids (MUFA), polyunsaturated fatty acids (PUFA), omega-3 (i.e., n-3) fatty acids, and omega-6 (i.e., n-6) fatty acids. Other cis and trans isomers of certain amino acids are also reported.

**Table 2:** Baki™ bean fatty acid profiles, representing samples (*N* = 9) from named varieties.

Fatty Acid	Minimum	Maximum	Mean ± SD	Mean ± SD (% total FA)
n-3 Fatty Acids	2.81	3.70	3.25 ± 0.30	39.54 ± 1.08
Alpha-Linolenic Acid (18:3 n-3)	2.79	3.66	3.22 ± 0.29	39.20 ± 1.11
n-9 Fatty Acids	1.94	2.75	2.34 ± 0.24	28.37 ± 0.97
Oleic Acid (18:1 cis-9)	1.90	2.68	2.29 ± 0.23	27.76 ± 0.95
n-6 Fatty Acids	1.28	1.64	1.45 ± 0.16	17.64 ± 1.01
Linoleic Acid (18:2 n-6)	1.26	1.59	1.43 ± 0.15	17.32 ± 1.04
Palmitic Acid (16:0)	0.67	0.93	0.78 ± 0.10	9.42 ± 0.39
n-6/n-3 ratio	0.40	0.50	0.45 ± 0.03	5.47 ± 0.63
Stearic Acid (18:0)	0.19	0.29	0.24 ± 0.03	2.96 ± 0.13
Vaccenic Acid (18:1 cis)	0.06	0.08	0.07 ± 0.01	0.79 ± 0.05
Behenic Acid (22:0)	0.05	0.07	0.06 ± 0.01	0.71 ± 0.05
Arachidic Acid (20:0)	0.03	0.04	0.04 ± 0.00	0.45 ± 0.02
Eicosenoic Acid (20:1 n-9)	0.03	0.04	0.03 ± 0.003	0.36 ± 0.01
Lignoceric Acid (24:0)	0.02	0.02	0.02 ± 0.003	0.23 ± 0.02
Conjugated Linoleic Acid (18:2)	0.01	0.03	0.01 ± 0.008	0.15 ± 0.08
Myristic Acid (14:0)	-	0.01	0.01 ± 0.004	0.13 ± 0.05
Margaric Acid (17:0)	0.01	0.01	0.01 ± 0.002	0.13 ± 0.01
Arachidonic Acid (20:4 n-6)	0.01	0.01	0.01 ± 0.002	0.12 ± 0.01
Pentadecanoic Acid (15:0)	–	0.01	0.01 ± 0.004	0.09 ± 0.05
Gamma Linolenic Acid (18:3 n-6)	–	0.01	0.00 ± 0.00	0.05 ± 0.07
Other Cis Isomers (18:1)	–	0.01	0.00 ± 0.00	0.04 ± 0.06
Margaroleic Acid (17:1)	–	0.01	0.00 ± 0.00	0.04 ± 0.05
Palmitoleic Acid (16:1)	–	0.01	0.00 ± 0.00	0.02 ± 0.04
Nonanoic Acid (9:0)	–	0.01	0.00 ± 0.00	0.01 ± 0.04

FA, fatty acids; −, Fatty acids with values below detection limit (<0.003 g) are omitted; fatty acids presented in descending order by mean value. Units reported as g/100 g sample, unless otherwise noted, on an edible portion dry matter (EPDM) basis.

Of the 45 fatty acids, 25 were present below the detection limit (0.003) and the content of each is therefore reported as <0.003 in Table 2. These include butyric acid (4:0), caproic acid (6:0), heptanoic acid (7:0) caprylic acid (8:0), capric acid (9:0), lauric acid (12:0), tridecanoic acid (13:0), myristoleic acid (14:1), 10-pentadecenoic acid (15:1), elaidic acid (18:1 trans-9), other trans isomers of 18:1, other cis and trans isomers of 18:2, nonadecanoic acid (19:0), eicosadienoic acid (20:2 n-6), eicosatrienoic acid (20:3 n-3), homo-gamma-linolenic acid (20:3 n-6), eicosapentaenoic acid (20:5 n-3), heneicosanoic acid (21:0), erucic acid (22:1 n-9), docosadienoic acid (22:2 n-6), docosapentaenoic Acid (22,5 n-3), docosahexaenoic acid (22,6 n-3), tricosanoic acid (23,0), and nervonic acid (24,1 n-9).

The remaining 20 fatty acids were present at levels above the detection limit (i.e > 0.003). Notable fatty acids, found to occur in the highest amounts, include, from highest to lowest amount, alpha-linolenic acid (18:3 n-3), oleic acid (18:1 cis-9) and linoleic acid (18:2 n-6). Of the n-3-6-9 fatty acids, n-3 had the highest content, followed by n-9 and n-6. The sainfoin varieties had a narrow range (0.01 g/100 g sample) in values for the ratio of n-6 to n-3 fatty acids (i.e., n-6/n-3).

**Figure 1:**
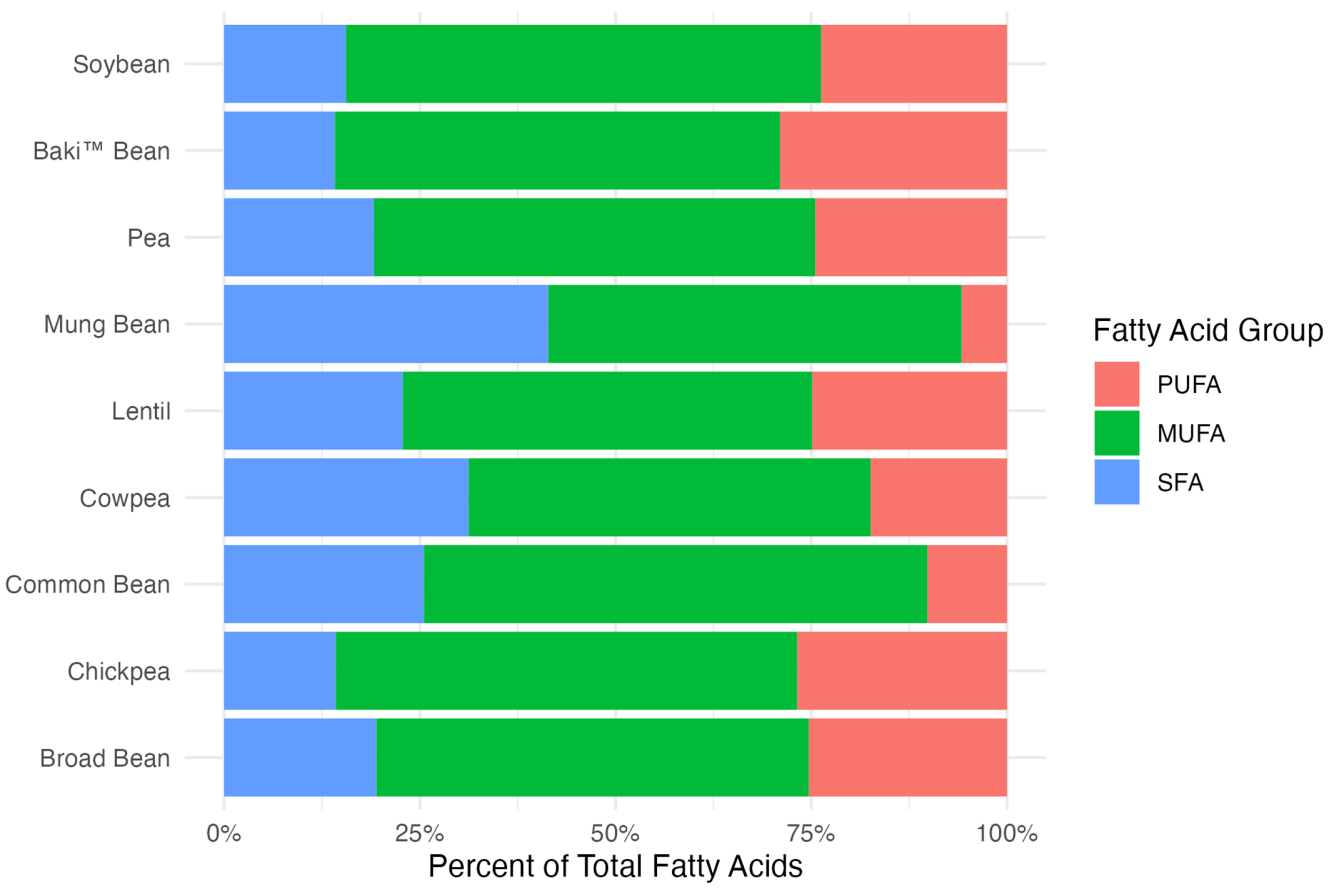
Fatty acid groups, including saturated fatty acids (SFA), monounsaturated fatty acids (MUFA), and polyunsaturated fatty acids (PUFA), shown for each crop species as the percentage of total fatty acids.

A comparison of the content of various fatty acids groups, including saturated fatty acids (SFA), monounsaturated fatty acids (MUFA), and polyunsaturated fatty acids (PUFA), between Baki™ bean, other pulse crops, and soybean is provided in Table 3 as g/100 g sample and in Figure 1 as a percent of total fatty acids. The fatty acid profile of sainfoin seeds was comprised primarily of MUFA, followed by PUFA, and is most comparable to broad bean and pea (Figure 1). The crops differed significantly with regards to SFA (*χ^2^*_8,52_ = 45.33; *p* < 0.001), MUFA (*χ^2^*_8,52_ = 46.66; *p* < 0.001), PUFA (*χ^2^*_8,52_ = 41.58; *p* < 0.001), and FA (*χ^2^*_8,52_ = 41.03; *p* < 0.001).

**Table 3:** Total content of each fatty acid group for each crop.

Crop	N	SFA	MUFA	PUFA	FA
**Baki bean™**	**9**	**1.17**^**a**^ **± 0.15**	**2.39**^**a**^ **± 0.24**	**4.68**^**a**^ **± 0.42**	**8.23**^**a**^ **± 0.79**
Broad Bean^1^	2	0.29^cd^ ± 0.08	0.38^cd^ ± 0.08	0.82^cd^ ± 0.31	1.50^bc^ ± 0.45
Chickpea^1^	6	0.65^a^ ± 0.10	1.22^a^ ± 0.32	2.68^ab^ ± 0.16	4.55^a^ ± 0.37
Common Bean^1^	10	0.37^bc^ ± 0.07	0.15^d^ ± 0.03	0.92^c^ ± 0.15	1.43^bc^ ± 0.22
Cowpea^1^	4	0.56^a^ ± 0.06	0.31^bc^ ± 0.14	0.92^bc^ ± 0.10	1.80^ab^ ± 0.27
Lentil^1^	7	0.24^d^ ± 0.05	0.26^c^ ± 0.07	0.55^d^ ± 0.10	1.04^c^ ± 0.20
Mung Bean^1^	3	0.55^ab^ ± 0.12	0.08^d^ ± 0.01	0.71^cd^ ± 0.21	1.34^bc^ ± 0.34
Pea^1^	10	0.31^cd^ ± 0.04	0.40^b^ ± 0.10	0.90^c^ ± 0.22	1.60^b^ ± 0.34
Soybean^2^	1	3.15^a^	4.82^a^	12.31^a^	27.77^a^

FA, fatty acids; S, saturated; MU, monounsaturated; PU, polyunsaturated. Units reported as g/100 g sample, edible portion dry matter (EPDM) basis. Within each column, values that share a letter are not statistically different (alpha = 0.05).

1 FAO (35).

2 USDA ARS Food Data Central.

### 3.2 Amino acid profiles

Essential amino acid (EAA) profiles are provided in Table 4. The crop species differed significantly with regard to content of histidine (*χ^2^*_8,52_ = 35.55, *p* < 0.001), isoleucine (*χ^2^*_8,52_ = 35.04, *p* < 0.001), leucine (*χ^2^*_8,52_ = 35.97, *p* < 0.001), lysine (*χ^2^*_8,52_ = 35.04, *p* < 0.001), methionine (*χ^2^*_8,52_ = 35.37, *p* < 0.001), threonine (*χ^2^*_8,52_ = 36.77, *p* < 0.001), tryptophan (*χ^2^*_8,52_ = 34.99, *p* < 0.001), and valine (*χ^2^*_8,52_ = 35.89, *p* < 0.001). No difference was found for phenylalanine content (*χ^2^*_8,52_ = 15.54, *p* < 0.0494).

**Table 4:** Essential amino acid content of each crop.

Crop	*N*	Histidine	Isoleucine	Leucine	Lysine	Methionine	Phenylalanine	Threonine	Tryptophan	Valine
**Baki bean™**	**9**	**1411**^**a**^ **± 77**	**1258**^**a**^ **± 81**	**2322**^**a**^ **± 136**	**1982**^**a**^ **± 112**	**568**^**a**^ **± 42**	**1324.73**^**a**^ **± 78.06**	**1288**^**a**^ **± 63**	**392**^**a**^ **± 13**	**1544**^**a**^ **± 93**
Broad Bean^1^	2	716^ab^ ± 23	1115^ab^ ± 35	1995^ab^ ± 64	1720^abc^ ± 57	182^b^ ± 5.8	1170^a^ ± 42	954^ab^ ± 31	238^bc^ ± 7.6	1230^abc^ ± 42
Chickpea^1^	6	632^b^ ± 74	933^bc^ ± 49	1652^b^ ± 100	1478^c^ ± 114	292^ab^ ± 53	1293^a^ ± 74	825^b^ ± 71	224^c^ ± 20	946^c^ ± 45
Common Bean^1^	10	687^b^ ± 47	1000^abc^ ± 137	1893^ab^ ± 200	1573^bc^ ± 161	263^ab^ ± 59	1285^a^ ± 147	1064^a^ ± 66	276^ab^ ± 36	1273^ab^ ± 210
Cowpea^1^	4	823^ab^ ± 31	1073^ab^ ± 107	1803^ab^ ± 168	1635^abc^ ± 76	397^a^ ± 43	1353^a^ ± 104	943^ab^ ± 41	278^ab^ ± 28	1189^abc^ ± 196
Lentil^1^	7	681^b^ ± 103	1163^a^ ± 78	2106^a^ ± 107	1974^a^ ± 108	235^b^ ± 13	1320^a^ ± 94	937^b^ ± 80	261^abc^ ± 29	1401^a^ ± 74
Mung Bean^1^	3	662^b^ ± 59	656 ^c^ ± 141	1687^b^ ± 224	1383^c^ ± 121	239^ab^ ± 59	1150^a^ ± 157	910^b^ ± 87	244^bc^ ± 24	1069^bc^ ± 139
Pea^1^	10	605^b^ ± 68	1002^abc^ ± 110	1784^b^ ± 198	1756^ab^ ± 192	243^ab^ ± 26	1175^a^ ± 136	924^b^ ± 112	237^bc^ ± 34	1175^abc^ ± 132
Soybean^2^	1	1210^ab^	2174^a^	365^a^	2985^a^	603^a^	2341^a^	1948^a^	652^a^	2238^a^

Values reported as mean value ± standard deviation. Units reported as mg/100 g sample, edible portion dry matter (EPDM) basis. Within each column, values that share a letter are not statistically different (alpha = 0.05).

1 FAO (35).

2 USDA ARS Food Data Central.

Nonessential amino acid (NEAA) profiles are provided in Table 5. The crops differed significantly with regards to alanine (*χ^2^*_8,52_ = 37.26, *p* < 0.001), arginine (*χ^2^*_8,52_ = 43.75, *p* < 0.001), aspartic acid (*χ^2^*_8,52_ = 35.59, *p* < 0.001), cystine (*χ^2^*_8,52_ = 40.47, *p* < 0.001), glycine (*χ^2^*_8,52_ = 40.94, *p* < 0.001), glutamic acid (*χ^2^*_8,52_ = 36, *p* < 0.001), proline (*χ^2^*_8,52_ = 32.24, *p* < 0.001), serine (*χ^2^*_8,52_ = 38.06, *p* < 0.001), tyrosine (*χ^2^*_8,52_ = 31, *p* < 0.001).

**Table 5:** Nonessential amino acid content of each crop.

Crop	*N*	Alanine	Arginine	Aspartic Acid	Cystine	Glutamic Acid	Glycine	Proline	Serine	Tyrosine
**Baki bean™**	**9**	**1360**^**a**^ **± 73**	**3938**^**a**^ **± 398**	**3677**^**a**^ **± 240**	**492**^**a**^ **± 23**	**6222**^**a**^ **± 478**	**1703**^**a**^ **± 73**	**1624**^**a**^ **± 108**	**1769**^**a**^ **± 118**	**1098**^**a**^ **± 60**
Broad Bean^1^	2	1105^bc^ ± 35	2615^ab^ ± 78	2980^ab^ ± 99	333^ab^ ± 11	4655^ab^ ± 148	1150^abc^ ± 42	1105^ab^ ± 35	1300^ab^ ± 42	881^ab^ ± 28
Chickpea^1^	6	969 ^c^ ± 51	2097^bc^ ± 256	2487^b^ ± 133	412^ab^ ± 198	4007^b^ ± 329	862^d^ ± 45	1,036 ^ab^ ± 148	1188^b^ ± 101	663^b^ ± 52
Common Bean^1^	10	1107^bc^ ± 64	1475^d^ ± 91	2951^ab^ ± 167	137^c^ ± 48	3918^b^ ± 225	1050^c^ ± 104	1101^ab^ ± 280	1411^a^ ± 97	732^ab^ ± 169
Cowpea^1^	4	1075^bc^ ± 75	1763^cd^ ± 270	2736^b^ ± 317	161^c^ ± 57	4258^ab^ ± 300	987^cd^ ± 140	1128^ab^ ± 85	1152^b^ ± 182	747^ab^ ± 106
Lentil^1^	7	1410^a^ ± 148	2140^bc^ ± 123	3363^a^ ± 258	306^ab^ ± 56	5063^a^ ± 530	1197^ab^ ± 64	1339^a^ ± 103	1321^ab^ ± 94	773^ab^ ± 67
Mung Bean^1^	3	1197^ab^ ± 115	1620^cd^ ± 165	2850^ab^ ± 363	187^bc^ ± 20	4247^ab^ ± 591	1733^a^ ± 145	1147^ab^ ± 116	1653^a^ ± 140	635^b^ ± 89
Pea^1^	10	1102^bc^ ± 125	2179^b^ ± 237	2885^ab^ ± 318	308^ab^ ± 51	4269^ab^ ± 481	1103^bc^ ± 122	1029^b^ ± 115	1172^b^ ± 153	785^ab^ ± 107
Soybean^2^	1	2112^a^	3478^ab^	5638^a^	722^a^	8685^a^	2074^a^	2624^a^	2600^a^	1698^b^

Values reported as mean value ± standard deviation. Units reported as mg/100 g sample, edible portion dry matter (EPDM) basis. Within each column, values that share a letter are not statistically different (alpha = 0.05).

1 FAO (35).

2 USDA ARS Food Data Central.

A comparison of the content of various groups of amino acids (e.g., EAA, NEAA) between sainfoin, pulse crops, and soybean is provided in Table 6. The crops differed significantly with regards to content of total amino acids (*χ^2^*_8,52_ = 36.19, *p* < 0.001), EAA (*χ^2^*_8,52_ = 34.77, *p* < 0.001), branched-chain amino acids (BCAA) (*χ^2^*_8,52_ = 36.4, *p* < 0.001), sulfur amino acids (SAA) (*χ^2^*_8,52_ = 36.9, *p* < 0.001), aromatic amino acids (AAA) (*χ^2^*_8,52_ = 23.68, *p* < 0.01), NEAA (*χ^2^*_8,52_ = 37.12, *p* < 0.001), and the ratio of essential to nonessential amino acids (EAA/NEAA) (*χ^2^*_8,52_ = 40.66, *p* < 0.001).

**Table 6:** Total content of various amino acids groups by each crop.

Crop	*N*	AA	EAA	BCAA	SAA	AAA	NEAA	EAA/NEAA
**Baki bean**^**TM**^	**9**	**33976**^**a**^**± 2160**	**12090**^**a**^**± 628**	**5124**^**a**^**± 301**	**1060**^**a**^**± 63**	**2423**^**a**^**± 137**	**21885**^**a**^**± 1535**	**0.55**^**c**^**± 0.01**
Broad Bean^1^	2	25444^ab^ ± 827	9321^ab^ ± 308	4340^ab^ ± 141	515^abc^ ± 17	2051^ab^ ± 71	16123^ab^ ± 520	0.58^bc^ ± 0
Chickpea^1^	6	21996^b^ ± 1484	8276^b^ ± 446	3531^b^ ± 187	704^a^ ± 215	1957^ab^ ± 118	13720^b^ ± 1057	0.60^b^ ± 0.02
Common Bean^1^	10	23195^b^ ± 1317	9313^ab^ ± 902	4165^ab^ ± 521	401^c^ ± 87	2017^ab^ ± 280	13882^b^ ± 724	0.67^a^ ± 0.07
Cowpea^1^	4	23495^ab^ ± 2004	9492^ab^ ± 604	4065^ab^ ± 396	558^ab^ ± 44	2100^ab^ ± 201	14002^b^ ± 1441	0.68^a^ ± 0.03
Lentil^1^	7	26991^a^ ± 1015	10079^a^ ± 607	4670^a^ ± 236	541^ab^ ± 66	2094^ab^ ± 138	16912^a^ ± 782	0.60^bc^ ± 0.05
Mung Bean^1^	3	23267^ab^ ± 2648	7999^b^ ± 973	3411.66^b^ ± 500	426^bc^ ± 76	1785^ab^ ± 246	15268^ab^ ± 1678	0.52^c^ ± 0.01
Pea^1^	10	23732^ab^ ± 2648	8900^ab^ ± 987	3961^b^ ± 439	551^ab^ ± 71	1960^ab^ ± 241	14831^ab^ ± 1662	0.60^bc^ ± 0.00
Soybean^2^	1	47431^a^	17800^a^	8062^a^	1326^a^	4038^a^	29631^a^	0.60^bc^

AA, amino acids; EAA, essential AA; BCAA, branched-chain AA (leucine, isoleucine, valine); SAA, sulfur AA (methionine, cystine); AAA, aromatic AA (phenylalanine, tyrosine); NEAA, nonessential AA; EAA/NEAA, ratio of essential to nonessential AA. Values reported as mean value ± standard deviation. Units reported as mg/100 g sample, edible portion dry matter (EPDM) basis. Within each column, values that share a letter are not statistically different (alpha = 0.05).

1 FAO (35).

2 USDA ARS Food Data Central.

### 3.3 Amino acid daily requirements

**Figure 2:**
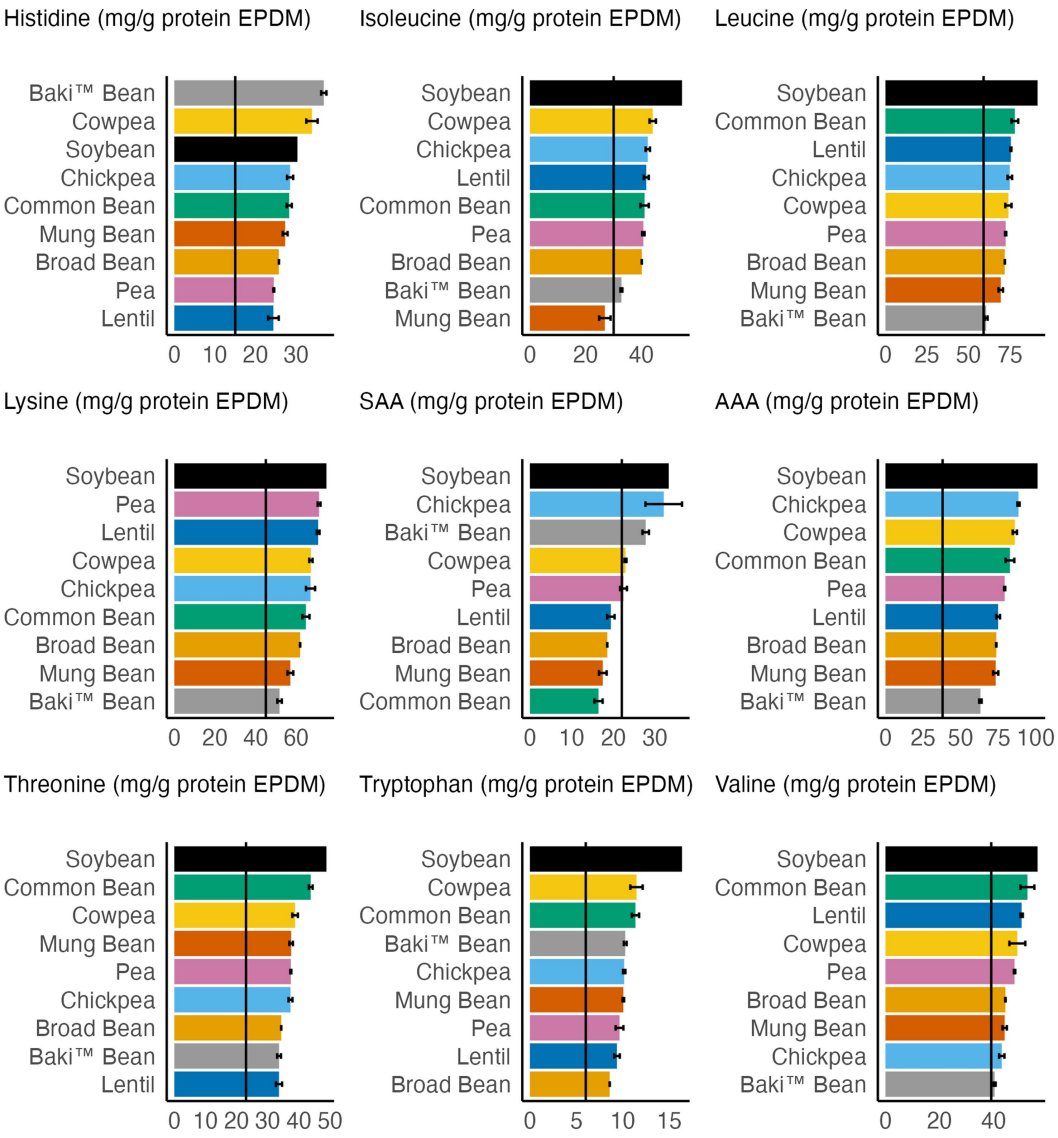
Comparison of essential amino acid content of each crop to adult daily requirements (solid, black vertical line) (FAO/WHO/UNU, 2007). Error bars represent one standard error of the mean.

A comparison of EAA content to adult (>18 years old) daily requirements is provided in Figure 2. Mean values for all crops met the daily requirements for histidine, leucine, lysine, AAA, threonine, tryptophan, and valine. The mean value for mung bean failed to meet isoleucine and SAA daily requirements. Additionally, lentil, broad bean, mung bean, and common bean mean values failed to meet SAA daily requirements. While the pea mean value (22.4 mg/g protein, dry matter) met the SAA requirement, within one standard error of the mean (2.6 mg/g protein, dry matter) pea fails to meet this requirement.

Since the values are reported as mg/g protein in Figure 2, compared to g/100 g sample as in Table 4, the maximum and minimum values vary. For example, soybean had the maximum value for lysine content (g/100 g sample) and mung bean had the minimum value (Table 4), compared to soybean having the maximum value for lysine content (mg/g protein) and Baki™ bean having the minimum value (Figure 2). Furthermore, Baki™ bean had the minimum value for leucine, lysine, and AAA, followed by mung bean and broad bean in each instance, and valine, followed by chickpea and mung bean. Lentil had the minimum value for histidine, followed by pea and broad bean; mung bean had the minimum value for isoleucine, followed by Baki™ bean and broad bean; common bean had the minimum value for SAA, followed by mung bean and broad bean; lentil had the minimum value for threonine, followed by Baki™ bean and broad bean; and broad bean had the minimum value for tryptophan, followed by lentil and pea. Soybean had the maximum value for each EAA, except for histidine in which Baki™ bean, followed by cowpea and soybean, had the maximum value and SAA, in which chickpea, followed by Baki™ bean and soybean, had the maximum values.

## 4 Discussion

### 4.1 Favorable fatty acid profile

A limited number of studies provide empirical data for fatty acid profiles of seed samples from sainfoins, representing various species with the *Onobrychis* genus. For instance, Tarasenko et al. (27) analyzed seed samples of *O. arenaria*, Bagci et al. (30) analyzed *O. major*, *O. altissima*, *O. hypargyrea*, and *O. huetiana*, Bakoglu et al. (31) analyzed *O. fallax*, Wijekoon et al. (46) analyzed *O. viciifolia* (cv. Melrose), and Kaplan et al. (32) analyzed 20 different genotypes of *O. viciifolia*. In general, fatty acid profile of sainfoin seeds is primarily composed of alpha-linolenic acid, oleic acid (18:1 n-9), linoleic acid (18:2 n-6). However, among these species, the fatty acid profiles vary with regard to the predominant fatty acids. Bagci et al. (30) found higher values for linoleic acid (31.5–51.8%) and Bakoglu et al. (31) found higher values for oleic acid (52.56%), with each representing the most abundant fatty acid. This is compared to our results, where we found linolenic acid to be the most abundant fatty acid (39.12%), which agrees with Tarasenko et al. (27) (41.41%) and Kaplan et al. (32) (33.15–41.22%). Moreover, we also found oleic acid as the second most abundant, which agrees with the results of Tarasenko et al. (27), Bagci et al. (30), and Kaplan et al. (32). Wijekoon et al. (46) report comparable amounts of linolenic (25.7%) and oleic acid (25.2%), followed by linoleic acid (20.0%). Interestingly, we detected myristic acid in our samples (0.000–0.133%), as did Bagci et al. (30) (0.2–0.9%), Wijekoon et al. (46) (0.30%), and Kaplan et al. (32) (0.00–0.36%), while Tarasenko et al. (27) and Bakoglu et al. (31) found this fatty acid to be absent in *O. arenaria* and *O. fallax*, respectively. Additionally, it appears that the presence or absence of erucic acid (22:1 n-9) varies across species. We found erucic acid content to be below the detection limit. Erucic acid content was also found to be absent in *O. fallax* (31), *O. major*, *O. altissima*, and *O. hypargyrea* (30), but was detected in *O. arenaria* (0.24%) (27) and *O. huetiana* (1.6%) (30). A lack of erucic acid is favorable, because this fatty acid is regulated in Europe, the U.S., Australia, and New Zealand to maintain content in oils below 5% (Europe) and 2% by weight, respectively (47, 48). Sainfoin fatty acids appear to be predominantly unsaturated, based on our results (85.8%), and those reported by Wijekoon et al. (46) (68.6%) and Kaplan et al. (32) (85.72–89.50%). While we did not test for this specifically, Kaplan et al. (32) showed that genotype had a significant effect on both *O. viciifolia* fat and fatty acid content. This indicates that genetic diversity may exist and could be used during the breeding process to influence fatty acid content and composition. In lupin (*Lupinus albus*), genotype and genotype-by-environment interaction have been shown to significantly impact total FA, MUFA, PUFA, and n-6/n-3 ratio, while genotype had a significant impact on oil content (49). Studies investigating how genotype, environment, management, and their interactions impact the fatty acid profiles of sainfoin seeds should be conducted. This information will be valuable for producers and breeders interested in identifying sources of variation and the extent of variation in fatty acid content and composition.

Linoleic (18:2 n-6) and alpha-linolenic acid (18:3 n-3) cannot be synthesized by the body and must be acquired through the diet. Therefore, these fatty acids are defined as essential fatty acids (50). We found the essential fatty acids alpha-linolenic acid and linoleic acid to be the most abundant and third most abundant fatty acids in Baki™ bean Because humans lack the enzymes to convert between n-6 and n-3 fatty acids, the proportion of n-6 to n-3 fatty acids (i.e., n-6/n-3 ratio) is of particular concern for nutritionist and dietitians who advocate for an appropriate balance to optimize health, growth, and development (50). While a ratio of 1/1 to 4/1 is recommended, most Western diets are considerably imbalanced with a ratio 15/1–16.7/1 (51). With a mean value of 0.447 (2/5 ratio), Baki™ bean appears to have a lower n-6/n-3 ratio compared to other pulses and is most similar to the *Phaseolus* group, including navy bean (0.91), kidney bean (0.81), and black bean (0.90). This group is contrasted by much higher ratios for chickpea (19.67) and broad bean (14.59) (52). Excessive intake of n-6 fatty acids and insufficient intake of n-3 can lead to several chronic diseases, such as cardiovascular disease, diabetes, and several cancers, which are prevalent in Western societies, and increasingly prevalent in developing countries where diets are being transformed by the influence of Western consumption patterns and the availability of cheap, energy dense foods (51, 53–56). These foods include meat and dairy products from corn and soy fed animals, high n-6 vegatable oils (e.g., corn, soy, sunflower, cottonseed), and processed foods comprised primarily of corn and soy (57). Even though Baki™ bean and other pulse crops generally have lower lipid and fatty acid content compared to oil seeds and oil legumes (e.g., soybean and peanut), they can still serve as an important source of fatty acids in human diets. In the context of increasing the intake of pulses in diets, fatty acid content and composition becomes increasingly important. For example, in a review of fatty acid profiles of selected pulses, data presented by Hall et al. (58) (% out of total fat) shows that linoleic acid is the primary fatty acid for chickpea (57%) and lentil and pea (48%). Linolenic acid content is highest for kidney (46%), great northern (43%), pinto (43%), navy (40%), mung (36%), and black (36%) beans. Conversely, linolenic acid content is lowest for lentil (12%), pea (10%), lupin (9%), and chickpea (2%). Finally, oleic acid is the most abundant fatty acid for lupin, and the second most abundant for chickpea, lentil, mung bean, and pea. Therefore, pulses can differ in their fatty acid profile, especially with regard to fatty acids essential to human health. For Baki™ bean, the composition of the fatty acid profile indicates that it can provide beneficial fatty acids for human nutrition, due to the high proportion of polyunsaturated to monounsaturated and saturated fatty acids, the relatively high content of the essential fatty acid alpha-linolenic acid (18:3 n-3), and high proportion of n-3 fatty acids compared to n-6 fatty acids, especially compared to other pulse crops. Specifically, Baki™ bean had higher SFA content than the values reported for broad bean, common bean, lentil, and pea, higher MUFA content than content than broad bean, common bean, cowpea, lentil, mung bean, and pea, higher PUFA content than broad bean, common bean, cowpea, lentil, mung bean, and higher FA content than broad bean, common bean, lentil, mung bean, and pea (Table 3). Enhancing these components could be a target of biofortification, or the breeding of crops to increase nutritional value (59, 60), as has been proposed for chickpea (61).

### 4.2 Amino acid profiles of sainfoin seeds

Tarasenko et al. (27) also report data for *O. arenaria* amino acid content. It is worth noting that the values they report are not on a dry matter basis, and that the seeds analyzed had a reported moisture content of 8.5%. Adjusting the values they report to a dry matter basis (i.e., 0% moisture) and unit to mg/100 g sample allows for a more direct comparison. We found higher total AA content (33,975.65 versus 29,442.62). When comparing the content of EAAs, we found higher content for histidine (1,411.24 versus 1,016.39), the sum of leucine and isoleucine (3,580.03 versus 3,245.90), lysine (1,982.03 versus 1,737.70), the sum of phenylalanine and tyrosine (2,423.05 versus 2,163.93), proline (1,624.00 versus 1,497.27), tryptophan (392.25 versus 142.08), valine (1,544.10 versus 1,398.91), and slightly higher content for threonine (1,287.62 versus 1,267.76). We found lower content for the sum of methionine and cystine (1,060.39 versus 1,191.26). Several factors could have contributed to these differences, such as the differing species and varying production methods.

Few additional studies provide insights into *Onobrychis* spp. amino acid content. Ditterline (28) found amino acid composition of sainfoin seeds to be comparable to soybean meal, and our amino acid profile results are comparable to those reported in Table 28 in their study. Baldinger et al. (29) analyzed the content of a limited number of amino acids, including lysine, tryptophan, methionine and cysteine content. They report a slightly lower amount of lysine. Futhermore, we found a slightly higher amount of tryptophan, as well as methionine and cysteine, compared to their results. Interestingly, Baldinger et al. (29) found a ratio for Lysine:Met+Cys:Threonine:Tryptophan in sainfoin of 100:56:60:17, which they claim to be close to the ideal ratio of 100:60:65:18 recommended for piglets with 5–20 kg body weight (62). They also report that this ratio was higher than the ratio of reported for peas of 100:33:53:13 (63). Ultimately, their findings indicate that sainfoin seeds could be a viable option for inclusion in weanling pig diets 10–16% compared to peas or soybean cake.

In this study, Baki™ bean had higher content of each of the nine essential amino acids, except for methionine and phenylalanine, than chickpea and mung bean. Additionally, Baki bean™ had higher methionine content than broad bean and lentil, higher histidine content than common bean, lentil and pea, higher leucine content than pea, higher lysine content than common bean, higher threonine content than lentil and pea, and higher tryptophan content than broad bean and pea (Table 4). Considering nonessential amino acid content Baki bean™ had higher alanine content than broad bean, chickpea, common bean, cowpea, and pea, higher arginine content than, chickpea, common bean, cowpea, lentil, mung bean, and pea, and higher aspartic acid content than chickpea and cowpea. Additionally, Baki™ bean had higher cystine content than common bean, cowpea, and mung bean, higher glutamic acid content than common bean and chickpea, higher glycine content than common bean, chickpea, cowpea, and pea, higher proline content than pea, higher serine content than chickpea, cowpea, and pea, higher tyrosine content than chickpea and mung bean (Table 5). Finally, Baki™ bean had higher total amino acid content than common bean and chickpea, higher essential amino acid content than chickpea and mung bean, higher branched chain amino acid content than chickpea, mung bean, and pea, higher sulfur amino acid content than common bean and mung bean, and higher nonessential amino acid content than chickpea, common bean, and cowpea. Sainfoin bean had a lower ratio of essential to nonessential acids than chickpea, common bean, and cowpea (Table 6).

### 4.3 Potential complete protein

In addition to their possible uses and value in animal diets, pulses are regarded as an important source of protein in human diets. Traditional human diets have relied on complimentary combinations of cereals and pulses as a solution to satisfying protein and amino acid requirements. Typically, the low-lysine content of cereals is supplemented by the content in pulses and the low-SAA content of pulses are supplemented by the content in cereals (5, 64, 65). Therefore, this strategy helps to mitigate the risk of limiting amino acid content in the diet. We define limiting amino acid content as insufficient content of a single essential amino acid, or multiple amino acids, when compared to the respective adult daily requirements established by the World Health Organization (WHO) and FAO (66). The crops analyzed in this study had a narrow range in values for the ratio of essential amino acids to nonessential amino acids, indicating that this level of analysis is not as informative as considering the content of individual amino acids. Our analysis of the FAO pulses data set shows that mung bean, lentil, broad bean, and common bean can have limiting essential amino acid content, especially for the sulfur amino acids (SAA) methionine and cysteine. Comparatively, we did not identify any limiting essential amino acids in the Baki™ bean samples analyzed. This indicates that seeds from sainfoins may provide a complete protein source with respect to satisfying essential amino acid requirements. Future studies are necessary to not only corroborate the results we provide regarding the amino acid profiles, but to also investigate how different combinations of genotypes, environments, management practices and processing techniques influence amino acid content and composition. Even though the ability of legumes to fix nitrogen through symbiotic associations with specific species of *Rhizobium* bacteria is believed to enhance the stability of seed protein content across environments (67), significant effects of environment and genotype-by-environment on seed protein content have been shown for *Vigna stipulacea* (68), *Lens culinaris* (69), *Cicer arietinum* (70). Moreover, genotype can also impact seed protein content. For example, Baptista et al. (71) found that certain bean and cowpea genotypes had amino acid scores close to meeting requirements. Moreover, in a study of cooked pulses, Nosworthy et al. (72) report amino acid scores (content/reference requirement) for the sulfur amino acids (methionine + cysteine) ranging from a limiting value of 0.59 for split red lentils and split green peas to a value of 1.08 for chickpeas that exceeds requirements. Conversely, chickpeas had the lowest score for tryptophan (0.61), compared to the highest score found for black beans (0.95). Scores for lysine, the amino acid typically limiting cereals, ranged from 1.16 for red kidney beans to 1.40 for whole green lentils. Sulfur fertilization and later harvest time can increase cysteine and methioine content, as has been shown for lentils (73). As with fatty acid content and composition, this information will be valuable for producers and breeders focused on improving sainfoin protein quality.

## 5 Conclusion

This study builds on evidence supporting the potential of sainfoin as a novel pulse crop. We quantified the amino acid and fatty acid profiles of Baki™ bean, representing seeds from named sainfoin varieties grow in the western US by commercial seed producers, and made comparisons to pulse crops using data reported by the FAO. Baki™ bean amino acid and fatty acid content was found to be higher than certain pulse crops. Baki™ bean fatty acids were primarily polyunsaturated, compared to monounsaturated and saturated fatty acids. The fatty acid profile was primarily composed of n-3 fatty acids, followed by n-9 fatty acids and then n-6 fatty acids. We found the essential fatty acid linolenic acid (18:3 n-3) to be the most abundant fatty acid, followed oleic acid (18:1 cis-9), and the essential fatty acid linoleic acid (18:2 n-6). When comparing essential amino acid content to adult daily requirements, Baki™ bean met the requirements for each amino acid. Moreover, we found that Baki™ bean, in addition to chickpea, soybean, cowpea, and pea, met sulfur amino acid requirements, which are typically limiting for pulses, as evidenced by lentil, broad bean, mung bean, and common bean failing to meet requirements. Future studies are required to further investigating the promising amino acid and fatty acid profiles found in this study for Baki™ bean.

## Data availability statement

The original contributions presented in the study are included in the article/Supplementary material, further inquiries can be directed to the corresponding author.

## Author contributions

EC: Conceptualization, Data curation, Formal analysis, Investigation, Project administration, Software, Validation, Visualization, Writing – original draft, Writing – review & editing. SB: Conceptualization, Methodology, Project administration, Writing – review & editing. MŞ: Conceptualization, Writing – review & editing. TP: Conceptualization, Funding acquisition, Project administration, Resources, Writing – review & editing. BS: Conceptualization, Data curation, Formal analysis, Funding acquisition, Investigation, Methodology, Project administration, Resources, Software, Supervision, Validation, Writing – original draft, Writing – review & editing.

## Footnotes

^1^https://www.montanaseeds.com/about-us

^2^https://www.nrcs.usda.gov/plantmaterials/mtpmctn12043.pdf
